# Association between pre-existing cardiovascular risk factors and post-acute sequelae of COVID-19 in older adults

**DOI:** 10.23938/ASSN.1103

**Published:** 2025-02-14

**Authors:** Guilherme José Silva Ribeiro, André de Araújo Pinto, Gabriela Corrêa Souza, Emilio Hideyuki Moriguchi

**Affiliations:** 1 Department of Nutrition Graduate Program in Nutritional Science Viçosa Brazil; 2 State University of Roraima Health Sciences Center Boa Vista Brazil; 3 School of Medicine Department of Nutrition and Graduate Program in Food, Nutrition, and Health Porto Alegre Brazil; 4 School of Medicine Graduate Program in Cardiology and Cardiovascular Sciences Porto Alegre Brazil

**Keywords:** Heart Disease Risk Factors, COVID-19, Post-Acute COVID-19 Syndrome, Older Adults, Epidemiology, Factores de Riesgo Cardiovascular, COVID-19, Secuelas Post Agudas de COVID-19, Adulto Mayor, Epidemiología

## Abstract

**Background:**

The long-term health impacts of COVID-19, including post-acute sequelae of SARS-CoV-2, remain insufficiently explored, especially concerning pre-existing cardiovascular risk factors in older adults. This study examines the association between these risk factors and post-acute sequelae of SARS-CoV-2 in this population.

**Methods:**

A retrospective study of Brazilian adults aged ≥ 60 years assessed the persistence of post-acute sequelae of SARS-CoV-2 three months after infection in 2020. Cardiovascular risk factors (obesity, smoking, high blood pressure, diabetes mellitus, hypercholesterolemia, and chronic kidney disease) were analyzed in relation to sequelae and adjusting for sociodemographic variables. Data were obtained from the Department of Epidemiological Surveillance in Roraima, Brazil.

**Results:**

Of the 1,322 participants (55% female; mean age 70.4 years, SD = 7.87), 61.7% (95% CI: 59.1-63.9) reported at least one post-acute sequelae of SARS-CoV-2 at the three-month follow-up. The likelihood of post-acute sequelae of SARS-CoV-2 was significantly higher in participants with diabetes mellitus (OR = 4.39; 95% CI: 3.42-5.66), tobacco use (OR = 3.93; 95% CI: 2.47-6.23), hypertension (OR = 3.62; 95% CI: 2.73-4.78), or hypercholesterolemia (OR = 3.58; 95% CI: 2.80-4.59). Chronic kidney disease (OR = 2.28; 95% CI: 1.59-3.25) and obesity (OR = 1.83; 95% CI: 1.28-2.61) were less strongly associated.

**Conclusions:**

Pre-existing cardiovascular risk factors are linked to a higher likelihood of long-term COVID-19 sequelae in adults aged ≥ 60 years old. Preventing and managing these factors are crucial for reducing the long-term effects of COVID-19, particularly during a pandemic.

## RESUMEN

**Fundamento:**

Los desafíos de salud a largo plazo de la COVID-19, incluidos sus efectos post-agudos (PASC), están poco estudiados en relación a los factores de riesgo cardiovascular (FRCV) preexistentes. Nuestro objetivo es analizar la asociación entre los FRCV preexistentes y el PASC en personas adultas mayores.

**Material y métodos:**

Este estudio retrospectivo incluyó a personas brasileñas de 60 años o mayores. Se evaluó la persistencia de PASC tres meses después de la infección por SARS-CoV-2 en 2020. Su relación con distintos FRCV como obesidad, tabaquismo, hipertensión arterial, diabetes mellitus, hipercolesterolemia y enfermedad renal crónica, se ajustó por todas las variables sociodemográficas disponibles. Los datos se obtuvieron de un sistema de control interno del Departamento de Vigilancia Epidemiológica en Roraima, Brasil.

**Resultados:**

El 61,7% (IC95%: 59,1-63,9) de las 1.322 personas estudiadas (55% mujeres y edad promedio 70,4 años, DE = 7,87) presentó al menos un PASC tras tres meses de seguimiento. La probabilidad de PASC fue mayor en adultos mayores con diabetes mellitus (OR = 4,39; IC95%: 3,42-5,66), tabaquismo (OR = 3,93; IC95%: 2,47-6,23), hipertensión (OR = 3,62; IC95%: 2,73-4,78) o hipercolesterolemia (OR = 3,58; IC95%: 2,80-4,59), La asociación fue menor con enfermedad renal crónica (OR = 2.28; IC95%:1.59-3.25) y con obesidad (OR = 1.83; IC95%: 1.28-2.61).

**Conclusiones:**

Los factores de riesgo cardiovascular preexistentes en personas de 60 años o mayores se asocian con una mayor probabilidad de presentar COVID-19 de larga duración, tras ajustar por las variables sociodemográficas disponibles. La prevención y el manejo de estos factores de riesgo son esenciales para reducir los efectos a largo plazo de COVID-19, especialmente durante una pandemia.

## INTRODUCTION

Since the emergence of the COVID-19 pandemic in 2020, the global epidemiological landscape of severe acute respiratory syndrome (SARS-CoV-2) has reached devastating proportions, significantly affecting public health and overburdening healthcare systems^1^. As the pandemic evolved, a substantial proportion of patients began reporting persistent symptoms following the initial viral infection^2^. Furthermore, the pandemic took new directions with the emergence of more transmissible SARS-CoV-2 variants, such as Delta and Omicron^3^.

Post-COVID-19 symptoms -referred to by the scientific and medical community as long COVID, post-COVID syndrome, or post-acute sequelae of SARS-CoV-2 (PASC)- are defined as symptoms persisting for more than 30 days after initial SARS-CoV-2 infection^4^. A previous study identified over 100 post-COVID-19 symptoms reported by patients who had recovered from the infection, with common complaints including dyspnea, palpitations, chest tightness, and chest pain^2^. A recent systematic review also found that individuals experiencing post-COVID-19 symptoms reported a significantly worse health-related quality of life^5^. These persistent challenges may place additional strain on healthcare systems and create new hurdles for medical practitioners.

As of today, more than 775 million cases of SARS-CoV-2 infection have been confirmed globally, with at least 34,000 new cases reported during the last week of June 2024, underscoring the ongoing persistence of the disease^6^. Among those affected, various pre-existing cardiovascular risk factors (CVRF), including hypertension, diabetes, obesity, dyslipidemia, and heart and lung diseases, have been linked to worsened health outcomes following SARS-CoV-2 infection^4^. Additionally, advanced age has been identified as a risk factor for the development of severe forms of the disease, highlighting the need for special attention to older adults^7^. Notably, a well-conducted study demonstrated that older age is also a risk factor for the development of PASC. However, the relationship between pre-existing comorbidities and persistent symptoms of COVID-19 in older adults remains insufficiently explored^8^.

In Brazil, vaccination efforts have resulted in a decline in the incidence of cases, hospitalizations, and deaths. However, the emergence of new SARS-CoV-2 variants continues to pose a public health threat^9^^,^^10^. Despite the progression in vaccination, vaccine hesitancy -partly driven by ideological factors- remains prevalent. It is critical to highlight that COVID-19 vaccination has been shown to reduce the likelihood of developing post-COVID-19 symptoms by 50% among individuals who received the initial two vaccine doses^11^. Therefore, healthcare professionals must prioritize and actively promote vaccination, especially among older adults, regardless of the presence of comorbidities. Failure to vaccinate leaves this vulnerable population at greater risk of both SARS-CoV-2 infection and the subsequent development of PASC.

Given the evolving nature of COVID-19, it is crucial to assess the incidence of post-acute sequelae resulting from persistent SARS-CoV-2 infection^12^. Studies focused on this issue may help mitigate the challenges faced by affected individuals and address gaps in our understanding of associated comorbidities, pathophysiology, and the long-term management of COVID-19. Furthermore, research on the long-term clinical management of older patients is essential to assist healthcare professionals in identifying those at greater risk of developing long COVID-19 symptoms. The aim of this study was to analyze the association between pre-existing CVRF and PASC in adults aged 60 years and older.

## MATERIALS AND METHODS

### Study design and context

This retrospective cohort study was conducted in the state of Roraima, Brazil in November 2022. It was based on health monitoring records of older adults who were infected with SARS-CoV-2 during 2020.

Roraima, the smallest state in Brazil (223,644.534 km²), is located in the northenmost region of the country, an area characterized by limited and challenging land access. The state has a population of 636,707 inhabitants, with a population density of 2.85 inhabitants per km^2^. Approximately 65% of the population reside in the capital city of Boa Vista. The estimated population of older adults aged 60 years or older is 50,000 and their average life expectancy is 71.8 years^13^. The first reported case of COVID-19 in Roraima was reported on March 21, 2020.

In 2020, a team of healthcare professionals conducted follow-up monitoring of older adults diagnosed with SARS-CoV-2. Initial contact was made through phone calls during the first weeks after diagnosis, followed by a second follow-up three months later. Using a standardized form provided by the Department of Health, older adults or their family members reported the presence or absence of symptoms. The information was recorded on the form during the phone call and subsequently entered into the virtual internal control system maintained by the Department of Epidemiological Surveillance of the Roraima State Health Secretariat. In the final phone call, personal and sociodemographic information related to COVID-19, comorbidities, and the presence of PASC were also collected.

We selected records of individuals who met the following criteria: 1) older adults aged 60 years or older, 2) a confirmed diagnosis of SARS-CoV-2 through PCR testing using nasal swabs, and 3) availability of complete information from the follow-up by healthcare professionals during the first year of the pandemic.

Access to the data was granted after receiving the necessary authorizations from the appropriate regulatory bodies. Authorization was provided on September 12, 2022, by the State Secretary of Health (Protocol number: 17201.004319/2022.64).

### Sample Size Calculation

According to data from the Department of Epidemiological Surveillance, 4,194 older adults were diagnosed with SARS-CoV-2 in 2020. During an exploratory analysis of the data, it was discovered that a significant amount of information had not been entered into the Health Department´s internal control system. Given the lack of information on long-term COVID in older Brazilian adults, a pilot study was conducted to explore the situation and inform the sample size calculation. Using the same follow-up instrument from the Department of Health, 104 older adults (who were excluded from the final analysis) completed a questionnaire on PASC. Of these, 60% reported experiencing at least one symptom that persisted for more than three months.

The sample size was estimated based on the number of older adults diagnosed with SARS-CoV-2 in 2020 and the prevalence of symptoms reported in the pilot study. The following parameters were used: a 95% confidence level (CI), a prevalence of 50% (for the evaluating multiple outcomes), a tolerable margin of error of 4%, and a design effect correction factor *(deff)* of 2. To account for possible losses and to ensure an adequate sample size, the final sample size was increased by 20%. As a result, the minimum required sample size was determined to be 1,260 participants.

### Study variables

The dependent variable in this study was the persistence of PASC, assessed during the final follow-up contact made three months after SARS-CoV-2 infection. Participants were asked whether they had experienced specific symptoms, including pain in the chest, chest tightness, palpitations, dyspnea (shortness of breath), and leg swelling. Older adults who reported experiencing at least one of these symptoms at the end of the follow-up period were classified as having PASC. The healthcare team responsible for participant monitoring underwent training on assessment procedures and adhered to medical guidelines to accurately differentiate these symptoms. According to the Health Department responsible for monitoring the data, none of the participants reported any signs or symptoms prior to the diagnosis of SARS-CoV-2 infection.

The pre-existing CVRF included in the health assessment forms for older adults were as follows: 1) arterial hypertension (defined by sustained blood pressure levels ≥140/90 mm Hg); 2) diabetes mellitus (fasting plasma glucose ≥126 mg/dL [7.0 mmol/L] after an overnight fast of at least 8 hours or hemoglobin A1c ≥6.5%); 3) obesity (body mass index >30 kg/m²); 4) chronic kidney disease (CKD) (reduced kidney function with a glomerular filtration rate [GFR] <60 mL/min/1.73m² confirmed after three months, and/or an albumin-to-creatinine ratio [ACR] ≥30 mg/g); 5) hypercholesterolemia (total cholesterol ≥200 mg/dL); and 6) smoking status, assessed using the question: *Do you smoke?* with response options “*yes*” or “*no*”. The Ministry of Health of Brazil, through the National Cancer Institute, operationally defines smoking as the regular and continuous consumption of tobacco products such as cigarettes, cigars, or pipes.

Sociodemographic information collected included sex (male, female) and age (recorded in complete years), which were later categorize into the following age groups (60-69, 70-79, ≥80). Skin color/race was classified according to a national classification system (yellow, white, brown, black, indigenous), and place of residence was recorded as either urban (capital) or rural (interior regions). The education level of the participants was documented, with the following categories: no schooling, ≤8 years of education, or >8 years of education.

### Statistical analysis

Age is presented as the mean with standard deviation (SD), while categorical variables as absolute and relative frequencies, along with their 95% CI. Pearson´s chi-square test was used to assess potential interactions between comorbidities (obesity, smoking, arterial hypertension, diabetes mellitus, hypercholesterolemia, CKD) and sex. The prevalence of PASC, both overall and for each specific symptom, is presented along with the corresponding 95% CI. Binary logistic regression models (both crude and adjusted) were employed to evaluate the association between pre-existing comorbidities and the presence of PASC, with odds ratios (OR) and their corresponding 95% CI. Regardless of the p-value in the crude models, all comorbidities were adjusted for covariates (sex, age, skin color, education, and location) in the adjusted model. All data were analyzed using IBM SPSS Statistics software (Version 20.0; IBM Corp., Armonk, NY, USA). A p-value of <0.05 was considered statistically significant.

## RESULTS

The final sample consisted of 1,322 older adults who tested positive for SARS-CoV-2 in 2020, with 55.0% of participants being female. The mean age was 70.4 years (SD = 7.87). The majority were aged between 60 and 69 years old and had no formal education. Additionally, most participants identified as having brown skin color and resided in the state capital. The most prevalent CVRF among the participants were hypertension (75.3%), hypercholesterolemia (54.4%), and diabetes mellitus (51.9%) (Table 1).

**Table 1:** General characteristics of adults aged ≥ 60 years old infected with SARS-CoV-2 (Roraima-BR, 2020)

Variable	Proportion (95% CI)
*Age (years)*	
mean (SD)	70.4 (7.87)
60-69	53.9 (51.1-56.1)
70-79	31.5 (29.1-33.6)
≥80	14.7 (12.9-16.3)
*Sex*	
Female	55.0 (52.4-57.3)
Male	45.0 (42.4-47.2)
*Education level*	
no education	47.2 (44.5-49.5)
≤8 years	29.9 (27.5-32.0)
>8 years	22.9 (20.7-24.8)
*Skin color*	
yellow	4.6 (4.3-4.8)
white	22.8 (20.6-24.7)
brown	45.2 (42.5-47.4)
black	16.8 (14.8-18.5)
indigenous	10.6 (9.0-12.0)
*Place of residence*	
interior	35.9 (33.3-38.1)
capital	64.1 (61.5-66.2)
*Comorbidities*	
obesity	14.0 (12.2-15.6)
smoking	11.6 (9.90-13.0)
hypertension	75.3 (73.0-77.2)
diabetes mellitus	51.9 (49.3-51.1)
hypercholesterolemia	54.4 (51.7-56.6)
chronic kidney disease	16.8 (14.8-18.5)

SD: standard deviation; CI: confidence interval.

After the three-month follow-up, more than half of the older adults (61.7%; 95% CI: 59.1-63.9) reported having experienced at least one PASC. The most frequently reported sequelae were palpitations (48.6%; 95% CI: 45.2-50.3) and chest tightness (45.7%; 95% CI: 43.1-48.0), followed by dyspnea (25.9%; 95% CI: 23.5-27.8), and chest pain (24.9%; 95% CI: 22.6-26.9). Leg swelling was less common with a prevalence of 12.0% (95% CI: 10.2-13.5).

Table 2 shows the association between pre- existing CVRF and the development of PASC. The crude analysis revealed that older adults with pre-existing CVRF had a significantly higher likelihood of developing PASC. In the adjusted model, after controlling for covariates, all CVRF remained statistically associated with the development of PASC (p <0.001). Older adults with diabetes mellitus, smoking, hypertension, and hypercholesterolemia exhibited the highest odds of developing PASC. Additionally, those with obesity and CKD also had increased odds, although to a lesser extent.

**Table 2:** Association between pre-existing cardiovascular risk factors and the development of post-acute sequelae of COVID-19 in older adults

Variables	Prevalence (%)	Logistic regression analysis OR (95%CI)	
		Crude	Adjusted^*^
*Obesity*			
no	60.2	1	1
yes	71.4	1.65 (1.17-2.32)	1.83 (1.28-2.61)
*Smoking*			
no	58.8	1	1
yes	83.8	3.61 (2.32-5.63)	3.93 (2.47-6.23)
*Hypertension*			
no	35.2	1	1
yes	70.5	4.40 (3.37-5.73)	3.62 (2.73-4.78)
*Diabetes Mellitus*			
no	42.5	1	1
yes	79.6	5.28 (4.15-6.74)	4.39 (3.42-5.66)
*Hypercholesterolemia*			
no	44.3	1	1
yes	76.4	4.06 (3.21-5.14)	3.58 (2.80-4.59)
*Chronic kidney disease*			
no	58.4	1	1
yes	78.4	2.59 (1.84-3.64)	2.28 (1.59-3.25)

OR: odds ratio; 95%CI: 95% confidence interval; *: model adjusted for sex, age (years), skin color, education level, and location.

## DISCUSSION

To the best of our knowledge, this is one of the first studies to examine the association between pre-existing CVRF and PASC specifically in older Brazilian adults. Our findings indicate that approximately six out of ten older adults experience persistent signs and/or symptoms after acute COVID infection. Furthermore, we found that the CVRF investigated are associated with an increased risk of PASC for at least three months after a SARS-CoV-2 diagnosis. Older adults with diabetes mellitus, smoking habits, hypertension, hypercholesterolemia, CKD, and obesity are at significantly increased risk for the sequelae we studied. One of the key contributions of this study is the evidence that, regardless of sex, age, education level, skin color, or place of residence, older adults with CVRF are at an increased risk of developing PASC.

Nearly two-thirds of older adults in this study experience post-acute COVID-19 sequelae lasting for more than three months. It is important to note that we cannot rule out the possibility that some of these symptoms may be related to pre-existing conditions prior to SARS-CoV-2 infection. A cohort study involving more than 800,000 participants showed that one or more symptoms persisted in older adults after the acute phase of COVID-19^4^. Similarly, another study that included adults with a mean age of 63 years reported a 63% increased risk of cardiovascular events over the course of 12 months, including dysrhythmias, heart disease, heart failure, thromboembolic disease, and other cardiac disorders^14^. These findings suggest that persistent symptoms of long-term COVID are likely to remain among older adults with comorbidities^2^. Consequently, multidisciplinary care is recommended to manage the heterogeneity and complexity of clinical manifestations that may affect the health of the geriatric population.

Obesity nearly doubled the risk of older adults experiencing PASC. Previous studies have similarly reported a correlation between a high BMI and an increased likelihood of experiencing prolonged symptoms after COVID-19^15^^,^^16^. The exact pathophysiological mechanisms underlying the association between obesity and post-COVID-19 syndromes remain unclear. However, it is known that obesity is a cardiometabolic risk factor that can contribute to inflammation and endothelial dysfunction. These processes may reduce the cardiometabolic reserve and lower the threshold for exertional symptoms^17^. It is hypothesized that chronic inflammation and immunometabolic processes contribute to the severe clinical course of acute SARS-CoV-2 infection and the development of long-term COVID symptoms^18^. Therefore, it is recommended that obese individuals affected by COVID-19 should undergo active health surveillance.

Our study demonstrates that smokers have nearly four times the likelihood of developing PASC compared to non-smokers, making smoking the second most significant risk factor. Although this relationship has not yet been specifically examined in older adult populations, it has been studied in other subgroups^19^^-^^21^. While the pathological mechanisms linking smoking to persistent symptoms of COVID remain unclear, it is well established that smoking weakens the immune system^22^. Additionally, smoking is a major risk factor for respiratory and cardiovascular outcomes, exacerbating the severity and mortality of both bacterial and viral infections by inducing mechanical and structural changes in the respiratory tract^23^^,^^24^. Given the association of smoking and SARS-CoV-2-related syndromes, further research is needed to clarify the underlying pathological mechanisms that lead to the development of long-term COVID symptoms. In light of this, public health interventions that focus on counseling and therapeutic support for smoking cessation should be implemented to help manage these persistent symptoms.

Older adults with hypertension were more than three times as likely to develop PASC, making hypertension the third most significant risk factor, consistent with findings from other studies^25^^-^^27^. From a molecular perspective, the association between hypertension and long-term COVID symptoms is plausible due to the systemic inflammatory response, which can lead to changes in vascular cells^28^. These mechanisms may result in renal and vascular dysfunction, elevating blood pressure and damaging vital organs. Given this, hypertension can exacerbate the chronic inflammatory response in patients with acute COVID-19, contributing to the development of long COVID^28^. This evidence is supported by a case-control study whose results found pre-existing hypertension to be associated with a higher number of long-term COVID symptoms compared to those without hypertension^29^. Consequently, extra care is needed for hypertensive patients, including proper medication management, blood pressure control, and promotion of a healthy lifestyle. Future studies should focus on subgroup analyses to explore whether blood pressure control or blood pressure levels influence post-COVID-19 sequelae.

Diabetes mellitus is the most significant pre- existing CVRF, increasing the likelihood of PASC development by 4.39-fold, in line with other studies^30^^-^^32^. It is well established that diabetes is associated with greater severity and worse progression of COVID-19^33^. The hypothesized mechanism for the presentation of post-COVID-19 symptoms involves endothelial damage, increased oxidative stress, and elevated pro-inflammatory cytokines^34^. These processes raise the risk of thromboembolic, pulmonary fibrosis, and acute respiratory distress syndrome, leading to more severe COVID-19 cases and, consequently, a higher risk of long-term COVID symptoms^35^.

Hypercholesterolemic older adults are more than three and a half times as likely to develop PASC compared to those without hypercholesterolemia. Our findings align with other studies that have reported this association^36^^,^^37^. It is theorized that elevated oxidized LDL concentration increases susceptibility to SARS-CoV-2 infection. A previous study showed lipid disorder SARS-CoV-2 infection, which compromised HDL function, reducing its anti-inflammatory and antioxidant effects^38^. Some thromboembolic events may be linked to increased lipoprotein levels during the course of the disease, which could, in turn, accelerate the progression of the formation of atherosclerotic lesions^39^. Furthermore, pro-inflammatory cytokines, such as IL-17A, IL-8, and IL-18 activate endothelial cells during atherogenesis, contributing to the chronic development of atherosclerotic plaques in post-COVID-19 patients^40^. These mechanisms have been documented in post-COVID-19 patients who experienced cardiovascular events due to clinically evident progression of coronary atherosclerosis^41^. As a result, the inflammatory activity within coronary atherosclerotic plaques increases the risk of rupture^42^. Further research is needed to investigate the relationship between hypercholesterolemia, the atherosclerotic process, and the persistence of long-term COVID symptoms.

Pre-existing CKD significantly associates with PASC, with affected individuals being more than twice as likely to develop it, consistent with a previous study^26^. Several studies have reported that the prevalence of CKD in patients infected with SARS-CoV-2^43^^,^^44^ ranges from 2% to 7%. Older adult with pre-existing CKD are at greater risk of developing the most severe form of SARS-CoV-2 infection^45^^,^^46^. The pathophysiological mechanisms linking CKD and post-acute COVID-19 syndromes remain unclear. Since CKD is associated with the development of cardiopulmonary diseases, which result in poor cardiorespiratory fitness, SARS-CoV-2 infection may trigger respiratory symptoms such as dyspnea and chest tightness^47^. Additionally, it is hypothesized that kidney damage caused by SARS-Cov-2 infection^48^ becomes a risk factor for cardiovascular events^49^. Nephrologists, cardiologists, and geriatricians should be especially vigilant with this population, as they are particularly susceptible to persistent symptoms of long-term COVID-19.

Although this study examines the persistence of symptoms following COVID-19, its retrospective design limits the ability to attribute the reported symptoms exclusively to SARS-CoV-2 infection, as pre-existing conditions may confound the results. One major limitation of this study is the use of non-specific symptoms, such as chest pain, palpitations, and dyspnea, to characterize long-term COVID. While these symptoms are common among patients with SARS-CoV-2, they are also frequently observed in various cardiovascular diseases, such as atrial fibrillation, coronary artery disease, and heart failure^50^. The lack of specific adjustment for these comorbidities may affect the accuracy of our findings, as similar symptoms could be attributed to pre-existing conditions rather than exclusively to SARS-CoV-2 infection. Furthermore, inflammatory biomarkers and other complementary tests were not analyzed, which could have provided additional insights into the pathophysiology of the persistent symptoms observed. Additionally, although a pilot study was conducted to estimate an adequate sample size and minimize selection bias, some degree of bias may still exist, as the sample may not fully represent the broader population of older adults. The retrospective design also introduces a certain level of subjectivity in the responses, particularly given the advanced age of participants or when responses were provided by someone other than the study subject. However, we minimized potential information bias through team training and the standardization of response variables. Finally, the possibility of symptom underreporting cannot be ruled out, as responses were provided to healthcare professionals and may reflect some degree of social desirability bias.

This is the first study to collect data on older adults with long COVID symptoms from an underexplored region of Brazil, providing valuable information for health management in this population. Additionally, the analyzed data are representative, as they were collected through the epidemiological monitoring system, which strengthens the external validity of the findings.

Our study shows that the prevalence of PASC in adults aged 60 years and older is high. All assessed CVRF (diabetes mellitus, smoking, hypertension, hypercholesterolemia, CKD, and obesity) significantly associate with an increased likelihood of PASC, even after adjusting for covariates such as age, sex, education level, skin color, and place of residence.

The CVRF examined in this study warrant further investigation to provide more evidence on the pathophysiological mechanisms involved in the observed associations. Future studies should focus on the duration of these symptoms and guide the management of long-term COVID-19, particularly among older adults, who appear to be frequently affected. These findings can help inform care strategies for older adults, both by public health agencies and by specialized professionals.

## Data Availability

The data of this study are available by petition to the corresponding author.
